# Integrative and complementary healthcare practices for hypertension: a summary of recommended clinical guidelines

**DOI:** 10.1590/S2237-96222025v34e20240844.en

**Published:** 2025-08-04

**Authors:** Marcus Tolentino Silva, Daniel Miele Amado, Paulo Roberto Sousa Rocha, Jorge Otávio Maia Barreto

**Affiliations:** 1 Universidade de Brasília, Departamento de Saúde Coletiva, Brasília, DF, Brazil; 2 Ministério da Saúde, Núcleo Técnico de Gestão da Política Nacional de Práticas Integrativas e Complementares em Saúde no SUS, Brasília, DF, Brazil; 3 Fundação Oswaldo Cruz, Gerência Regional de Brasília, Brasília, DF, Brazil

**Keywords:** Clinical Practice Guide, GRADE Approach, Integrative and Complementary Health Practices, Hypertension, Evidence- Based Policy, Guía de Práctica Clínica, Enfoque GRADE, Prácticas Integrativas y Complementarias en Salud, Hipertensión, Política Basada en Evidencia

## Abstract

**Objective:**

Identifying the main uses of integrative and complementary healthcare practices (ICPHPs) in managing systemic arterial hypertension in adults.

**Methods:**

Evidence summary. With eligibility criteria for the clinical guidelines from the Grading of Recommendations, Assessment, Development and Evaluation system and others that addressed ICPHPs hypertension, with no language or date restrictions. Medline, Embase and Scopus were the databases consulted. The Appraisal of Guidelines for Research and Evaluation II tool was used for risk of bias assessment, focusing on methodological rigor. The results were then summarized narratively, grouping the recommendations by outcome and analyzing commonalities and differences between the included guidelines.

**Results:**

Eight clinical guidelines were included in this evidence summary, selected from 560 records initially identified. The most common interventions were meditation, yoga, breathing techniques, tai chi and mindfulness, with a positive impact on blood pressure, stress control and patients’ quality of life. Most guidelines recommended these practices for hypertension control, based on moderate quality evidence with a strong recommendation.

**Conclusion:**

The results of this summary indicate that HCPs are effective and safe strategies in the complementary management for hypertension, suggesting a more holistic and less medicalized approach to managing the condition.

## Resumen

**Objetivo:**

Identificar los principales usos de las prácticas integrativas y complementarias en salud (PICS) en el manejo de la hipertensión arterial sistémica en adultos.

**Métodos:**

Se trata de una síntesis de evidencia. Los criterios de elegibilidad incluyeron guías clínicas que utilizan el sistema Grading of Recommendations, Assessment, Development and Evaluation y que abordaban PICS para la hipertensión, sin restricciones de idioma o fecha. Se consultaron las fuentes de información Medline, Embase y Scopus. El riesgo de sesgo se evaluó utilizando la herramienta Appraisal of Guidelines for Research and Evaluation II, centrándose en el rigor metodológico de las guías. La síntesis de los resultados se realizó de forma narrativa, agrupando las recomendaciones por desenlace y analizando convergencias y divergencias entre las guías incluidas.

**Resultados:**

Se incluyeron en esta síntesis de evidencia ocho guías clínicas, seleccionadas de 560 registros identificados inicialmente. Las intervenciones más comunes fueron meditación, yoga, técnicas de respiración, tai chi y mindfulness, con un impacto positivo en la presión arterial, el control del estrés y la calidad de vida de los pacientes. La mayoría de las guías recomiendan estas prácticas para el control de la hipertensión, basándose en evidencia de calidad moderada y con una recomendación fuerte.

**Conclusión:**

Los resultados de esta síntesis indican que las PICS son estrategias efectivas y seguras en el manejo complementario de la hipertensión, sugiriendo un enfoque más holístico y menos medicalizado en el tratamiento de la enfermedad.

**Ethical aspects**

Literature review based study, which does not require a Research Ethics Committee approval.:

## Introduction

Systemic hypertension represents a major challenge for Brazil’s Unified Health System (SUS), accounting for 19% of the system’s demands in 2017 (1). As the leading risk factor for cardiovascular disease and mortality in Brazil, hypertension has an impact on healthcare resources. In 2019, SUS alone (2) had an estimated expenditure of Int$ 581 million. Despite the reduction in age-adjusted mortality rates attributed to hypertension between 1990 and 2017, total deaths increased by 53%, largely due to demographic aging (1). SUS has been implementing strategies to deal with hypertension, especially by offering cost-free medication and multi-professional team support in primary care (2).

In this context, integrative and complementary health practices (ICHP) have been gaining ground in hypertension management and are considered valuable tools for fostering comprehensive health care. However, their introduction in SUS, which is mostly linked to traditional biomedicine, is still incipient (3). The National Policy for Integrative and Complementary Practices in SUS (NPICP) emerged as a cornerstone for the incorporation of ICHP into SUS, in line with health promotion guidelines (4). Among health professionals, especially nurses, there is a growing interest in ICHP, where 37% report having been trained and 14% use these practices in the management of hypertension (5). Techniques such as auriculotherapy and bloodletting – a practice derived from acupuncture – are often used to address contributing factors for hypertension such as anxiety and stress. These approaches can contribute to higher patient treatment compliance, as well as fostering a more holistic, patient-centered care.

Numerous factors influence the use of ICHP in hypertension management. Educational level, anxiety, gender and individual beliefs about the disease are important predictors for ICHP implementation (6,7). The most commonly used practices among hypertensive patients include use of medicinal plants, garlic and auriculotherapy (5,6,8). Although potential benefits are promising, the adoption of ICHP is still limited, which points to the need for further integration of such practices in SUS.

To ensure a robust evaluation of clinical guidelines, the GRADE (Grading of Recommendations, Assessment, Development and Evaluation) system has been widely adopted to assess the quality of evidence and recommendations (9,10). GRADE provides a systematic approach that considers the overall quality of studies, consistency of findings and potential biases, categorizing evidence into four levels (high, moderate, low and very low) and classifying the quality of recommendations as strong, weak or conditional (10). This approach integrates the best available literature with patient preferences and clinical experience.

The purpose of this study has been to determine the most widely recommended uses of ICHP in hypertension management in adults, based on a summary of available GRADE clinical guidelines.

## Methods

In this study, we present a summary based on a brief review for healthcare technology assessment (11). This brief review employs methodological simplifications to summarize the best available evidence in a short timeframe, and is especially useful in contexts that require timely responses to support clinical or healthcare policy decision-making. We have opted for a fast-track review in light of the political need to provide consistent and up-to-date evidence in the short term to subsidize discussions on ICHP in SUS. Among the changes implemented are the precise and objective definition of a research question, a focus on secondary studies (such as clinical guidelines), use of specific tools for critical appraisal, as well as an optimized data extraction and analysis process (carried out by a reviewer and revised by another, with a consensual resolution of discrepancies).

### Eligibility Criteria

The selection criteria we used in our summary for this study were clinical guidelines that: used the GRADE system to assess the quality of the evidence and recommendations; addressed the use of ICHP (apitherapy; aromatherapy; art therapy; ayurveda; biodance; bioenergetics; family constellation; chromotherapy; circular dance; geotherapy; hypnotherapy; homeopathy; laying on of hands; anthroposophical medicine/anthroposophy applied to healthcare; traditional Chinese medicine - acupuncture; meditation; music therapy; naturopathy; osteopathy; ozone therapy; medicinal plants - phytotherapy; chiropractic; reflexotherapy; reiki; shantala; integrative community therapy; flower therapy; social thermalism/crenotherapy; yoga) in hypertension management (blood pressure ≥130/80 mmHg or use of antihypertensive drugs) in adults (≥18 years); were available in any language, regardless of the date of publication. Choosing guidelines based on the GRADE system serves the robustness and transparency of method. The inclusion of guidelines without language or date restrictions aimed to broaden the scope and representativeness of the recommendations analyzed.

### Sources

We searched for clinical guidelines in Medline (via Pubmed), Embase and Scopus. These databases have a wide reach in healthcare and biomedical sciences and are recognized for their quality and scope of the journals indexed. Other databases, such as LILACS or Web of Science, were not included due to overlapping content and fewer exclusive clinical guidelines. The selection of these sources sought to optimize the efficiency of the search without compromising the scope of the results.

### Research Strategies

Table 1 shows our research strategy employed in identifying clinical guidelines on the use of ICHP in hypertension management. The strategy combined keywords associated with the words such as “hypertension”, “guidelines” and “GRADE system”, which were adjusted according to the specifics of each source. We used Boolean operators and descriptors specific to each platform in order to maximize sensitivity and specificity retrieving relevant information.

**Table 1:** Research strategy used in the selected databases and findings as of October 9, 2024

Source	Keywords researched	Results
Medline (via PubMed)	#1 “hypertension”[Mesh] OR (high blood pressure) OR (high blood pressures) #2 (guidelines as topic[MeSH:noexp] OR practice guidelines as topic[MeSH:noexp] OR Health Planning Guidelines[MeSH:noexp] OR practice guideline[MeSH:noexp] OR clinical protocols[MeSH:noexp] OR Consensus[MeSH:noexp] OR “Consensus Development Conference”[PTYP] OR “Consensus Development Conference, NIH”[PTYP] OR “Consensus Development Conferences as Topic”[MeSH:noexp] OR “Consensus Development Conferences, NIH as Topic”[MeSH:noexp] OR critical pathway[MeSH:noexp] OR (clinical[TIAB] AND pathway[TIAB]) OR (clinical[TIAB] AND pathways[TIAB]) OR (practice[TIAB] AND parameter[TIAB]) OR (practice[TIAB] AND parameters[TIAB]) OR algorithms[MeSH:noexp] OR care pathway[TIAB] OR care pathways[TIAB] OR guidance[TIAB] OR guideline*[TI]) #3 “GRADE system”[tiab] OR “GRADE methods”[tiab] OR “GRADE approaches”[tiab] OR “GRADE approach”[tiab] OR “Grading of Recommendations, Assessment, Development and Evaluation”[tiab] OR (“GRADE”[tiab] AND (“low”[tiab] OR “moderate”[tiab]OR “high”[tiab] OR “evidence”[tiab] OR “strong”[tiab] OR “weak”[tiab] “recommendation”[tiab])) #4 #1 AND #2 AND #3	172
Embase	#1 ‘hypertension’/exp OR (‘high blood pressure’) OR (‘high blood pressures’) #2 ‘practice guideline’/exp OR ‘practice guideline’ #3 ‘GRADE approach’/exp OR ‘GRADE approach’ #4 #1 AND #2 AND #3	115
SCOPUS	(TITLE-ABS-KEY (hypertension) OR TITLE-ABS-KEY (“‘high blood pressure’) OR TITLE-ABS-KEY (“‘high blood pressures’)) AND (TITLE-ABS-KEY(guidelines)) AND (TITLE-ABS-KEY(“GRADE system” OR “GRADE methods” OR “GRADE approaches” OR “GRADE approach” OR “Grading of Recommendations, Assessment, Development and Evaluation”))	262

### Screening criteria

We have selected our guidelines in two steps. Firstly, with the screening of studies’ titles and abstracts according to the predefined eligibility criteria by two independent reviewers. Discrepancies were settled by consensus. In our study’s second step, a reviewer assessed all potentially eligible records in detail, with a subsequent check by a second reviewer to ensure eligibility. A PRISMA flow chart was drawn up to illustrate the screening process.

### Data Gathering

The data from the included guidelines was extracted by a reviewer using a standardized spreadsheet, while a second reviewer verified the extracted information.

### Data Set

The data included: year of publication, country of origin, target population (type of hypertension), ICHP investigated, outcomes analyzed, use of ICHP recommendations, evidence quality (high, moderate, low or very low) and quality of recommendation (strong or weak).

### Assessment of studies’ risk of bias

In order to assess the risk of bias in the included guidelines, we relied on the “methodological rigor” section of the Appraisal of Guidelines for Research and Evaluation II (AGREE II) tool (12). This section comprises eight separate items which were assessed individually by a reviewer and verified by another. 

The reviewers classified the answers as “yes”, “partially” or “no”, considering: (i) the use of systematic methods in the research of evidence; (ii) clarity in criteria for the evidence assessment; (iii) explicit description of the strengths and limitations of the evidence included; (iv) transparency in issuing recommendations; (v) explicit consideration of benefits, risks and adverse health effects in the recommendations presented; (vi) a direct relationship between recommendations and evidence; (vii) peer reviewed process prior to publication; and (viii) documented procedures for periodically updating guidelines. Upon consideration, the discrepancies between the reviewers were resolved via consensus. Based on the assessment results, it was possible to measure the methodological rigor and overall quality of the included guidelines.

### Summary Methods

The included guidelines’ recommendations were analyzed in descriptively, considering the guidelines’ methodological quality and the quality of the evidence supporting each recommendation. They were grouped by outcome, with an emphasis on convergences and divergences amongst guidelines.

### Conclusion Assesment

Guideline confidence was determined by the AGREE II tool, assigning the highest priority to guidelines with a low risk of bias, i.e. those that answered “yes” to most of the methodological rigor criteria. Guidelines with the highest number of “yes” answers were classified as high confidence, while those with answers evenly split between “yes”, “partially” and “no” were classified as moderate confidence, and those with a predominance of “partially” and “no” answers were considered low confidence. The quality of the evidence and the strength of the recommendations were directly obtained from the GRADE system guidelines, thus allowing for a more precise interpretation of the recommendations. The recommendations were combined in a qualitative way, seeking balance based on a critical analysis of the available body of evidence.

## Results

**Figure 1:**
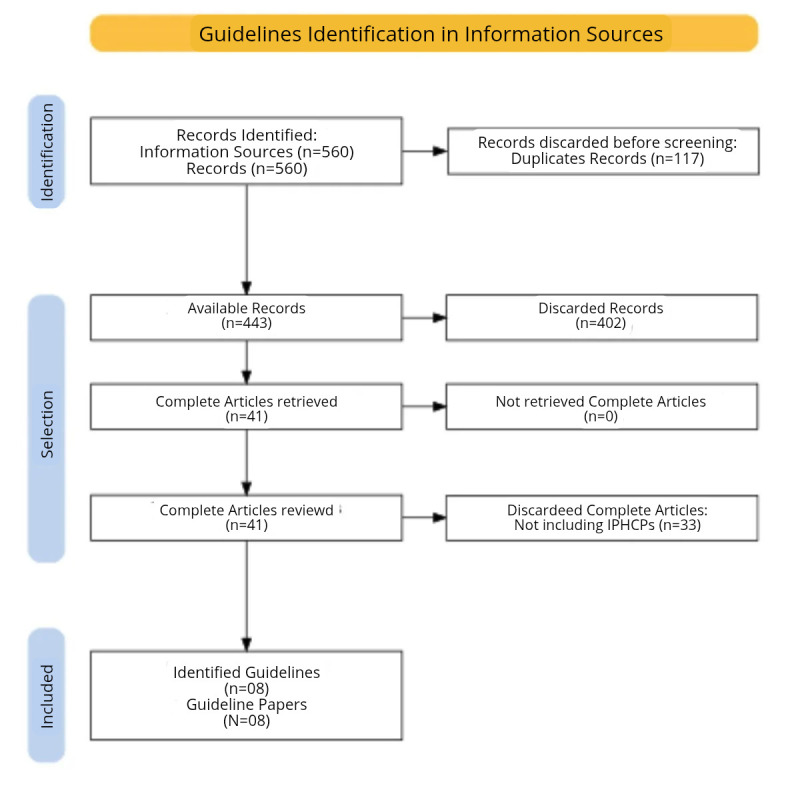
Flowchart identifying guidelines on hypertension and the use of integrative and complementary health practices (ICPHPs)

Results from each step of the hypertension guideline assessment for the evidence summary are presented in Figure 1. On October 9th, 2024, a total of 560 records had been identified in the databases. After removing 117 duplicates, 443 studies remained for screening. Out of these, 402 were discarded based on their titles and abstracts, resulting in 41 full-text studies. Upon assessment using the eligibility criteria, 33 studies were excluded, leaving 8 studies included in our summary (13-20).

Table 2 presents the clinical guidelines related to hypertension, comprising different geographical contexts and ICHP. These studies spanned accross different countries and contexts, such as Brazil, Spain, India, Japan, Taiwan, the United States and other low- and middle-income nations. The target population included adults with hypertension and different cardiovascular risks. Other types of hypertension (secondary, gestational, white coat or malignant) were not specified, suggesting a greater interest in primary hypertension. Treatments include transcendental meditation, yoga, relaxation techniques and biofeedback. The outcomes ranged from lower blood pressure to stress control and improved quality of life.

**Table 2:** Characterization of included clinical guidelines on hypertension

Author	Origin of the recommendation	Target Demographic	Integrative and complementary health practices	Results
Barroso 2021(13)	Brazil	Adults suffering from hypertension.	Transcendental Meditation. Guided Slow Breathwork. Music therapy. Spirituality and Religion.	Reduced systolic and diastolic blood pressure levels. Stress control. Improved overall life quality and treatment compliance.
Royo-Bordonada 2013 (14)	Spain	Patients at high cardiovascular risk and the general population.	Individualized Psychotherapy. Relaxation Techniques (meditation, breathwork, yoga, muscle relaxation).	Improved mood. Reduced stress.
Royo-Bordonada 2016 (15)	Spain	General population, patients at cardiovascular risk, diabetics and patients suffering from hypertension.	Self Help Groups.	Reduced cardiovascular risk.
Shah 2020 (16)	India	Adults suffering from hypertension.	Yoga. Meditation.	Reduced blood pressure. Improved general well-being. Reduced anxiety symptoms.
Umemura 2019 (17)	Japan	Patients with high blood pressure.	Yoga. Meditation. Biofeedback.	Reduction of stress-related hypertension. Stress control.
Unger 2020 (18)	Low and middle income countries	Adults suffering from hypertension.	Transcendental Meditation/mindfulness. Complementary or traditional Medicine (African, Chinese). Yoga.	Reduced blood pressure. Blood pressure control. Stress reduction.
Wang 2022 (19)	Taiwan	Adults suffering from hypertension.	Tai chi. Yoga. Meditation. Relaxing and Breathwork.	Reduced blood pressure. Reduced stress. Improved physical and mental well-being.
Whelton 2017 (20)	United States	Adults suffering from hypertension.	Yoga, meditation, biofeedback. Behavioral Therapy (guided breathwork and relaxation).	Reduced blood pressure. Reduced stress and improved overall well-being.

Table 3 presents a critical assessment of the clinical guidelines included in this summary. Most of the guidelines assessed used appropriate systematic methods for researching evidence. There has been significant variation in the presentation of evidence selection criteria. Most guidelines failed to provide a clear description of the evidence’s strengths and limitations. Only three guidelines provided a thorough and clear description of the methods used to formulate their recommendations. Most guidelines adequately considered health benefits, risks and side effects when formulating their recommendations. The connection between recommendations and evidence was explicitly demonstrated in almost all guidelines. Independent expert review was a common practice in the guidelines. The most recent guidelines provided clear procedures for their updating.

**Table 3:** Critical evaluation of the included clinical guidelines on hypertension

Author year	1^a^	2^b^	3^c^	4^d^	5^e^	6^f^	7^g^	8^h^	Guideline Trustworthiness
Barroso 2021(13)	Y^i^	P^j^	P	Y	Y	Y	Y	Y	High
Royo-Bordonada 2013 (14)	Y	P	Y	P	Y	Y	Y	P	High
Royo-Bordonada 2016 (15)	P	N^l^	N	N	Y	Y	N	N	Low
Shah 2020 (16)	P	P	P	Y	Y	Y	N	N	Moderate
Umemura 2019 (17)	N	P	P	P	Y	Y	N	N	Low
Unger 2020 (18)	Y	Y	P	Y	Y	Y	Y	Y	High
Wang 2022 (19)	Y	Y	P	Y	Y	Y	Y	Y	High
Whelton 2017 (20)	Y	Y	Y	Y	Y	Y	Y	Y	High

^a^ 1. Were systematic methods used to conduct the evidence search?; ^b^ 2. Are the criteria for selecting evidence clearly described?; ^c^ 3. Are the body of evidence’s strengths and limitations clearly described?; ^d^ 4. Are the methods for formulating recommendations clearly described?; ^e^ 5. Have the benefits, side effects and health risks been taken into account when formulating the recommendations?; ^f^ 6. Is there an explicit link between the recommendations and the supporting evidence?; ^g^ 7. Was the guideline externally reviewed by experts before publication?; ^h^ 8. Is a procedure for updating the guideline available?; ^i^ Yes; ^j^ Partially; ^l^ No.

Table 4 summarizes the recommendations for ICHP in the clinical guidelines for the management of hypertension. Guided slow breathing, yoga, mindfulness, tai chi and meditation are recommended strategies for controlling blood pressure in hypertensive individuals (quality of evidence: moderate; strength of recommendation: strong). Relaxation techniques such as meditation, yoga and breathing exercises have been found to improve stress and anxiety management (quality of evidence: moderate; strength of recommendation: strong). Approaches that promote well-being and autonomy, such as yoga, meditation, spirituality and religion, stood out for their impact on well-being ( evidence quality: moderate; strength of recommendation: strong). Music therapy, biofeedback and self-help groups also show promising results (evidence quality: low; strength of recommendation: weak).

**Table 4:** Summary of recommendations for the use of ICHP in hypertension clinical guidelines

Results	Recomendations	Evidence quality	Strength of recommendation	Trustworthyness
Blood pressure management	Guided slow breathwork, yoga and mindfulness have been recommended for blood pressure control in hypertensive individuals (13,16,18). Tai chi, yoga and meditation, as well as relaxation and breathing exercises, are also indicated as effective strategies (19).	Moderate	Strong	High
	Transcendental meditation is associated with better systolic and diastolic blood pressure management (13). Music therapy also shows promising results (13).	Moderate	Weak	High
	Insufficient evidence to confirm the long-term impact of yoga, meditation and biofeedback on blood pressure (20).	Low	Weak	High
	Complementary or traditional African and Chinese medicine are not recommended without robust clinical trials (18).	Very Low	Strong	High
Stress and anxiety management	Meditation, breathing exercises, yoga and muscle relaxation are effective in stress and anxiety management, especially in women (14,16)	Moderate	Strong	High
	Yoga, meditation and biofeedback have been suggested for stress management, although guided breathwork and relaxation are not recommended as primary interventions (17,20).	Low	Weak	Low
Overall quality of life	Self-help groups promote patients’ autonomy and overall quality of life (15).	Low	Weak	Low
	Spirituality and religion have been associated with improved blood pressure management and increased overall well-being (13). Yoga and meditation have been recommended for cardiovascular well-being and wellness routines (19).	Moderate	Strong	High

## Discussion

Our brief review’s findings indicate that clinical guidelines on the management of hypertension suggest that meditation, yoga, tai chi and breathing techniques are associated with improvement in blood pressure management and the general well-being of patients. Critical analysis of these guidelines revealed significant variations in the degree of clarity in the wording of the recommendations and in the description of the strengths and limitations of the evidence. Despite these differences, most of the guidelines were consistent in their assessment of health benefits and risks, highlighting the potential of ICHP as complementary approach to hypertension management.

The main limitations of this study include the exclusion of clinical guidelines that have not adopted the GRADE system, which may have led to the absence of relevant documents based on other evaluation systems. Method quality might vary among the guidelines included, due to inconsistencies in evidence selection criteria and in formulation of recommendations, which might occur in interpretation biases in the results biases into the interpretation of the findings (21,22). Our study might also be limited by omitting guidelines not indexed in the databases used, which might have resulted in a narrower scope of analysis. Publication bias is a critical concern in ICHP, as studies with positive results tend to get published more frequently than those with negative results, which can skew the assessment of the actual effectiveness of these measures (23).

Our summary points to the benefits of meditation, yoga, tai chi, breathing exercises and mindfulness in hypertension management, evidencing their positive impact on managing blood pressure and patients’ overall well-being. Meditation for hypertension patients over 60 and yoga for hypertension patients under 60 significantly reduce systolic and diastolic blood pressure (24). Both transcendental meditation and mindfulness-based stress reduction have been found to produce significant results in lowering blood pressure (25). Physiological factors for these benefits may be related to associated lower stress levels and improved self-regulation promoted by these activities, as well as relaxation and a reduced level of sympathetic activity, thereby resulting in better cardiovascular control (26, 27). It is worth noting that ICHP promote greater patient engagement with their own health, favoring a more holistic and less medicalized approach to healthcare, which can contribute to long-term adherence to treatment (28). These findings underline the importance of considering ICHP as a viable and complementary option to traditional pharmacological approaches in hypertension management.

ICHP’ potential to promote the well-being and autonomy of hypertensive patients is another relevant aspect highlighted in this review. Interventions such as yoga and meditation have been found to have a positive impact on hypertension management, as well as improving quality of life and general well-being (29,30). By allowing patients to actively participate in managing their healthcare, these practices promote a greater sense of autonomy and control over their condition, which can reduce sole dependency on pharmacological solutions (31,32). By fostering emotional and mental balance, these interventions can contribute to reducing symptoms associated with stress and anxiety, factors often correlated with hypertension (33, 34). This more comprehensive approach to treatment, involving physical, mental and spiritual aspects, is in line with a patient-centered healthcare paradigm, promoting not just symptom relief, but a transformation in lifestyle that improves both physical health and psychological well-being.

Further studies will require more rigorous and standardized clinical assessments of ICHP in hypertension. A systematic review shows that current clinical guidelines make little mention of these practices, pointing to an important gap in educating professionals and patients (35). We suggest that new clinical guidelines use systematic and transparent methods, preferably the GRADE system, to improve the overall quality of the recommendations. In Brazil, academic research indicates the positive impacts of techniques such as yoga, acupuncture and meditation on reducing blood pressure, but reinforces the need for more robust methods and larger samples (36). We therefore recommend that future research adopt rigorous designs, such as randomized clinical trials, with representative samples. Further studies should also investigate the impact of ICPH on long-term outcomes, such as treatment compliance, quality of life and cost-effectiveness, in order to provide comprehensive and sustainable evidence for clinical practice.

To sum up, this brief review indicates that HCPs have proven beneficial in managing hypertension, both in terms of managing blood pressure and improving patients’ overall well-being. Despite variations in methodological quality among guidelines, the evidence suggests that interventions such as meditation, yoga, tai chi and mindfulness are effective and safe strategies to complement conventional therapies. These findings underline PICS’ effectiveness as a promising approach to comprehensive hypertension care, with the potential to improve patients’ well-being and autonomy.

## Data Availability

No database and/or analytical key codes were produced in this research, apart from the illustrations already available in the manuscript.
