# Supplementary material for: Integrative and complementary healthcare practices for hypertension: a summary of recommended clinical guidelines
Source: Epidemiol Serv Saude. 2025 Aug 4;34:e20240844. doi: 10.1590/S2237-96222025v34e20240844.en (PMC12338984; doi:10.1590/S2237-96222025v34e20240844.en)
Supplement: Translation

# Translation: Práticas integrativas e complementares em saúde para hipertensão arterial: síntese de recomendações de diretrizes clínicas

**DOI:** 10.1590/S2237-96222025v34e20240844.pt

**Authors:** Marcus Tolentino Silva, Daniel Miele Amado, Paulo Roberto Sousa Rocha, Jorge Otávio Maia Barreto

**Affiliations:** 1 Universidade de Brasília, Departamento de Saúde Coletiva, Brasília, DF, Brasil; 2 Ministério da Saúde, Núcleo Técnico de Gestão da Política Nacional de Práticas Integrativas e Complementares em Saúde no SUS, Brasília, DF, Brasil; 3 Fundação Oswaldo Cruz, Gerência Regional de Brasília, Brasília, DF, Brasil

**Keywords:** Guia de Prática Clínica, Abordagem GRADE, Práticas Integrativas e Complementares em Saúde, Hipertensão, Política Informada por Evidências

## Abstract

**Objetivo:**

Identificar os principais usos das práticas integrativas e complementares em saúde (PICS) no manejo da hipertensão arterial sistêmica em adultos.

**Métodos:**

Trata-se de uma síntese de evidências. Os critérios de elegibilidade incluíram diretrizes clínicas que utilizam o sistema Grading of Recommendations, Assessment, Development and Evaluation e que abordaram PICS para hipertensão, sem restrições de idioma ou data. Foram consultadas as fontes de informação Medline, Embase e Scopus. O risco de viés foi avaliado com a ferramenta Appraisal of Guidelines for Research and Evaluation II, focando o rigor metodológico das diretrizes. A síntese dos resultados foi realizada de forma narrativa, agrupando as recomendações por desfecho e analisando convergências e divergências entre as diretrizes incluídas.

**Resultados:**

Foram incluídas oito diretrizes clínicas nesta síntese de evidências, selecionadas a partir de 560 registros inicialmente identificados. As intervenções mais comuns foram meditação, ioga, técnicas de respiração, tai chi e mindfulness, com impacto positivo sobre a pressão arterial, o controle do estresse e a qualidade de vida dos pacientes. A maioria das diretrizes recomendou essas práticas para o controle da hipertensão, baseando-se em evidências de qualidade moderada e com forte recomendação.

**Conclusão:**

Os resultados desta síntese indicam que PICS são estratégias eficazes e seguras no manejo complementar da hipertensão, sugerindo uma abordagem mais holística e menos medicalizada no tratamento da condição.

**Aspectos éticos**

Trata-se de estudo baseada em revisão da literatura, a qual dispensa aprovação por Comitê de Ética em Pesquisa.:

## Introdução

A hipertensão arterial sistêmica representa um desafio importante para Sistema Único de Saúde (SUS), com uma prevalência de 19%, em 2017 (1). Como principal fator de risco para doenças cardiovasculares e mortalidade no Brasil, a hipertensão tem impacto sobre os recursos de saúde. Em 2019, estima-se que Int$ 581 milhões foram gastos apenas no SUS (2). Apesar da redução das taxas de mortalidade ajustadas por idade atribuíveis à hipertensão entre 1990 e 2017, o total de mortes aumentou em 53%, em grande parte devido ao envelhecimento populacional (1). O SUS tem adotado estratégias para o enfrentamento da hipertensão, especialmente por meio da oferta de medicamentos gratuitos e do suporte de equipes multiprofissionais na atenção primária (2).

Nesse contexto, as práticas integrativas e complementares em saúde (PICS) têm ganhado espaço na gestão da hipertensão e são vistas como ferramentas valiosas para a promoção da saúde e o cuidado integral. No entanto, sua adoção no SUS, majoritariamente vinculado à biomedicina tradicional, ainda é incipiente (3). A Política Nacional de Práticas Integrativas e Complementares no SUS (PNPIC) surgiu como um marco para a incorporação de PICS no SUS, alinhando-se aos princípios de promoção da saúde (4). Entre os profissionais de saúde, especialmente enfermeiros, há um crescente interesse em PICS, com 37% relatando treinamento e 14% utilizando essas práticas no manejo da hipertensão (5). Técnicas como auriculoterapia e sangria – uma prática derivada da acupuntura – são frequentemente utilizadas para abordar a hipertensão arterial e fatores associados, como ansiedade e estresse. Essas abordagens podem contribuir para uma maior adesão ao tratamento, além de promoverem um cuidado mais holístico e centrado no paciente.

Diversos fatores influenciam o uso de PICS no manejo da hipertensão. Nível educacional, ansiedade, gênero e crenças sobre a doença são preditores importantes para a adoção de PICS (6,7). As práticas mais comuns entre pacientes hipertensos incluem o uso de plantas medicinais, alho e auriculoterapia (5,6,8). Embora os benefícios potenciais sejam promissores, a adoção das PICS ainda é limitada, destacando-se a necessidade de maior integração dessas práticas no SUS.

Para garantir a avaliação robusta de diretrizes clínicas, o sistema GRADE (Grading of Recommendations, Assessment, Development and Evaluation) tem sido amplamente adotado para avaliar a qualidade das evidências e a força das recomendações (9,10). O GRADE fornece uma abordagem sistemática que considera a qualidade dos estudos, consistência dos achados e potenciais vieses, categorizando as evidências em quatro níveis (alta, moderada, baixa e muito baixa) e classificando a força das recomendações como forte, fraca ou condicional (10). Essa abordagem integra a melhor literatura disponível com as preferências dos pacientes e a experiência clínica.

O presente estudo teve como objetivo identificar os principais usos recomendados das PICS no manejo da hipertensão em adultos, a partir de uma síntese de diretrizes clínicas GRADE disponíveis globalmente.

## Métodos

Este estudo apresenta uma síntese baseada em uma revisão rápida para avaliação de tecnologias em saúde (11). A revisão rápida utiliza simplificações metodológicas para sintetizar as melhores evidências disponíveis em um prazo reduzido, e é especialmente útil em contextos que exigem respostas oportunas para subsidiar decisões clínicas ou políticas em saúde. Optou-se pela revisão rápida devido à necessidade política de fornecer evidências consistentes e atualizadas em curto prazo para subsidiar as discussões sobre PICS no SUS. Entre as simplificações adotadas, estão a delimitação precisa e objetiva da pergunta de pesquisa, a priorização de estudos secundários (como diretrizes clínicas), o uso de ferramentas específicas para avaliação crítica, além da otimização no processo de extração e análise de dados (realizada por um revisor e revisada por outro, com resolução consensual das divergências).

### Critérios de elegibilidade

Os critérios de elegibilidade para inclusão de estudos nesta síntese foram: diretrizes clínicas que utilizassem o sistema GRADE para avaliar a qualidade da evidência e a força das recomendações; abordassem o uso de PICS (apiterapia; aromaterapia; arteterapia; ayurveda; biodança; bioenergética; constelação familiar; cromoterapia; dança circular; geoterapia; hipnoterapia; homeopatia; imposição de mãos; medicina antroposófica/antroposofia aplicada à saúde; medicina tradicional chinesa – acupuntura; meditação; musicoterapia; naturopatia; osteopatia; ozonioterapia; plantas medicinais – fitoterapia; quiropraxia; reflexoterapia; reiki; shantala; terapia comunitária integrativa; terapia de florais; termalismo social/crenoterapia; ioga) no tratamento da hipertensão (pressão arterial ≥130/80 mmHg ou uso de anti-hipertensivos) em adultos (≥18 anos); e estivessem disponíveis em qualquer idioma, independentemente da data de publicação. A escolha por diretrizes que adotam o sistema GRADE justifica-se pela robustez e transparência do método. A inclusão de diretrizes sem restrição de idioma ou data visou ampliar o escopo e a representatividade das recomendações analisadas.

### Fontes de informação

As diretrizes clínicas foram buscadas nas bases Medline (via Pubmed), Embase e Scopus. Essas bases têm ampla cobertura em saúde e ciências biomédicas e são reconhecidas pela qualidade e abrangência dos periódicos indexados. Outras bases, como LILACS ou Web of Science, não foram incluídas, devido à sobreposição de conteúdos e menor número de diretrizes clínicas exclusivas. A seleção dessas fontes buscou otimizar a eficiência da pesquisa sem prejuízo da abrangência dos resultados.

### Estratégia de busca

A Tabela 1 descreve a estratégia de busca utilizada para identificar diretrizes clínicas sobre o uso de PICS no tratamento da hipertensão. A estratégia combinou termos relacionados aos termos “hipertensão”, “diretrizes” e “sistema GRADE”, ajustados conforme a especificidade de cada fonte de informação. Foram utilizados operadores booleanos e descritores próprios de cada plataforma, visando maximizar a sensibilidade e a especificidade na recuperação de informação relevante.

**Tabela 1:** Estratégia de busca nas fontes de informação selecionadas e resultados obtidos em 9 de outubro de 2024

Fonte	Termos de busca	Resultados
Medline (via PubMed)	#1 “hypertension”[Mesh] OR (high blood pressure) OR (high blood pressures) #2 (guidelines as topic[MeSH:noexp] OR practice guidelines as topic[MeSH:noexp] OR Health Planning Guidelines[MeSH:noexp] OR practice guideline[MeSH:noexp] OR clinical protocols[MeSH:noexp] OR Consensus[MeSH:noexp] OR “Consensus Development Conference”[PTYP] OR “Consensus Development Conference, NIH”[PTYP] OR “Consensus Development Conferences as Topic”[MeSH:noexp] OR “Consensus Development Conferences, NIH as Topic”[MeSH:noexp] OR critical pathway[MeSH:noexp] OR (clinical[TIAB] AND pathway[TIAB]) OR (clinical[TIAB] AND pathways[TIAB]) OR (practice[TIAB] AND parameter[TIAB]) OR (practice[TIAB] AND parameters[TIAB]) OR algorithms[MeSH:noexp] OR care pathway[TIAB] OR care pathways[TIAB] OR guidance[TIAB] OR guideline*[TI]) #3 “GRADE system”[tiab] OR “GRADE methods”[tiab] OR “GRADE approaches”[tiab] OR “GRADE approach”[tiab] OR “Grading of Recommendations, Assessment, Development and Evaluation”[tiab] OR (“GRADE”[tiab] AND (“low”[tiab] OR “moderate”[tiab]OR “high”[tiab] OR “evidence”[tiab] OR “strong”[tiab] OR “weak”[tiab] “recommendation”[tiab])) #4 #1 AND #2 AND #3	172
Embase	#1 ‘hypertension’/exp OR (‘high blood pressure’) OR (‘high blood pressures’) #2 ‘practice guideline’/exp OR ‘practice guideline’ #3 ‘GRADE approach’/exp OR ‘GRADE approach’ #4 #1 AND #2 AND #3	115
SCOPUS	(TITLE-ABS-KEY (hypertension) OR TITLE-ABS-KEY (“‘high blood pressure’) OR TITLE-ABS-KEY (“‘high blood pressures’)) AND (TITLE-ABS-KEY(guidelines)) AND (TITLE-ABS-KEY(“GRADE system” OR “GRADE methods” OR “GRADE approaches” OR “GRADE approach” OR “Grading of Recommendations, Assessment, Development and Evaluation”))	262

### Processo de seleção

A seleção das diretrizes foi realizada em duas etapas. Primeiramente, dois revisores independentes realizaram a triagem de títulos e resumos dos estudos identificados, de acordo com os critérios de elegibilidade predefinidos. Divergências foram resolvidas por consenso. Na segunda etapa, os registros potencialmente elegíveis tiveram seus textos completos avaliados em detalhe por um revisor, com a verificação de um segundo revisor para garantir a elegibilidade. Um diagrama de fluxo PRISMA foi elaborado para ilustrar o processo de seleção

### Processo de coleta de dados

Um revisor extraiu os dados das diretrizes incluídas utilizando uma planilha eletrônica padronizada, enquanto um segundo revisor confirmou as informações extraídas.

### Lista de dados

Os dados coletados incluíram: ano de publicação, país de origem, população-alvo (tipo de hipertensão), PICS investigadas, desfechos analisados, recomendações sobre o uso das PICS, qualidade da evidência (alta, moderada, baixa ou muito baixa) e força da recomendação (forte ou fraca).

### Avaliação do risco de viés dos estudos

Para avaliar o risco de viés nas diretrizes incluídas, utilizou-se o domínio “rigor metodológico” da ferramenta Appraisal of Guidelines for Research and Evaluation II (AGREE II) (12). Esse domínio contém oito itens que foram avaliados individualmente por um revisor e confirmado por outro. Os revisores atribuíram as respostas “sim”, “parcialmente” ou “não”, considerando: (i) uso de métodos sistemáticos na busca das evidências; (ii) clareza nos critérios de seleção dessas evidências; (iii) descrição explícita dos pontos fortes e limitações das evidências incluídas; (iv) transparência na formulação das recomendações; (v) consideração explícita dos benefícios, riscos e efeitos adversos à saúde nas recomendações apresentadas; (vi) existência de uma relação clara entre recomendações e evidências; (vii) realização de revisão externa por especialistas antes da publicação; e (viii) existência documentada de procedimentos para atualização periódica das diretrizes. Após avaliação, as divergências entre os revisores foram resolvidas por consenso. A partir do julgamento obtido, foi possível determinar o rigor metodológico e a qualidade global das diretrizes incluídas.

### Métodos de síntese

A análise das recomendações das diretrizes incluídas foi conduzida de forma narrativa, levando em consideração a qualidade metodológica das diretrizes e a qualidade das evidências que embasam cada recomendação. As recomendações foram agrupadas por desfecho, com ênfase na identificação de convergências e divergências entre as diretrizes.

### Avaliação da certeza

A confiança nas diretrizes foi avaliada com base na aplicação da ferramenta AGREE II, conferindo maior peso às diretrizes com baixo risco de viés, ou seja, aquelas que responderam “sim” à maioria dos critérios de rigor metodológico. Diretrizes com maior número de respostas “sim” foram classificadas como de alta confiança, enquanto aquelas com respostas equilibradas entre “sim”, “parcialmente” e “não” foram classificadas como de confiança moderada, e as com predominância de respostas “parcialmente” e “não” foram consideradas de baixa confiança. A força das recomendações e a qualidade das evidências foram extraídas diretamente do sistema GRADE das diretrizes, permitindo uma interpretação mais precisa das recomendações. A combinação das recomendações foi feita de forma qualitativa, buscando equilíbrio a partir de uma análise crítica do conjunto de evidências disponíveis.

## Resultados

Os resultados de cada etapa do processo de seleção de diretrizes sobre hipertensão arterial para esta síntese de evidências estão descritos na Figura 1. Em 9 de outubro de 2024, foram identificados 560 registros nas fontes de informação. Após a remoção de 117 duplicações, restaram 443 estudos para triagem. Desses, 402 foram excluídos com base nos títulos e resumos, resultando em 41 estudos avaliados em texto completo. Após a aplicação de critérios de elegibilidade, 33 estudos foram excluídos, restando 8 estudos incluídos na síntese (13-20).

**Figura 1:** Fluxo de identificação das diretrizes sobre hipertensão e uso de práticas integrativas e complementares em saúde (PICS)

As diretrizes clínicas relacionadas à hipertensão, abrangendo diversos contextos geográficos e PICS, estão caracterizadas na Tabela 2. Os estudos englobaram diferentes países e contextos, como Brasil, Espanha, Índia, Japão, Taiwan, Estados Unidos e outras nações de baixa e média renda. A população-alvo incluiu adultos hipertensos e com diferentes riscos cardiovasculares. Outros tipos de hipertensão (secundária, gravidez, branca ou maligna) não foram especificados, sugerindo maior interesse pela hipertensão essencial. As intervenções incluem meditação transcendental, ioga, técnicas de relaxamento e biofeedback. Dentre os desfechos, destacam-se redução da pressão arterial, controle do estresse e melhora na qualidade de vida.

**Tabela 2:** Caracterização das diretrizes clínicas incluídas sobre hipertensão

Autor/ano	Contexto da diretriz	População-alvo	Práticas integrativas e complementares em saúde	Desfechos
Barroso 2021(13)	Brasil	Adultos com hipertensão arterial.	Meditação transcendental. Respiração lenta guiada. Musicoterapia. Espiritualidade e religiosidade.	Redução da pressão arterial sistólica e diastólica. Controle de estresse. Melhor qualidade de vida e adesão ao tratamento.
Royo-Bordonada 2013 (14)	Espanha	Pacientes com risco cardiovascular elevado e população em geral.	Terapias psicológicas individualizadas. Técnicas de relaxamento (meditação, respiração, ioga, relaxamento muscular).	Melhoria no estado de ânimo. Redução do estresse.
Royo-Bordonada 2016 (15)	Espanha	População em geral, pacientes com risco cardiovascular, diabetéticos e hipertensos.	Grupos de autoajuda.	Redução do risco cardiovascular.
Shah 2020 (16)	Índia	Adultos com hipertensão arterial.	Ioga. Meditação.	Redução da pressão arterial. Melhora do bem-estar geral. Redução de sintomas de ansiedade.
Umemura 2019 (17)	Japão	Pacientes com hipertensão arterial.	Ioga. Meditação. Biofeedback	Redução da hipertensão associada ao estresse. Controle do estresse.
Unger 2020 (18)	Países de baixa e média renda	Adultos com hipertensão arterial	Meditação transcendental/mindfulness. Medicinas complementares ou tradicionais (África, China). Ioga.	Redução da pressão arterial. Controle da pressão arterial. Redução do estresse.
Wang 2022 (19)	Taiwan	Adultos com hipertensão arterial.	Tai chi. Ioga. Meditação. Exercícios de relaxamento e respiração.	Redução da pressão arterial. Redução do estresse. Melhora do bem-estar físico e mental.
Whelton 2017 (20)	Estados Unidos	Adultos com hipertensão arterial.	Ioga, meditação, biofeedback. Terapias comportamentais (respiração guiada, relaxamento).	Redução da pressão arterial. Redução do estresse e melhora do bem-estar.

A avaliação crítica das diretrizes clínicas incluídas nesta síntese está apresentada na Tabela 3. A maioria das diretrizes avaliadas utilizou métodos sistemáticos adequados para a busca de evidências. Houve variação importante na descrição dos critérios de seleção de evidências. A maioria das diretrizes falhou em fornecer uma descrição clara dos pontos fortes e limitações das evidências. Apenas três diretrizes forneceram uma descrição completa e clara dos métodos utilizados para a formulação de suas recomendações. A maior parte das diretrizes consideraram adequadamente os benefícios, riscos e efeitos colaterais à saúde na formulação de suas recomendações. A relação entre recomendações e evidências foi explicitamente demonstrada em quase todas as diretrizes. A revisão externa por especialistas foi uma prática comum nas diretrizes. As diretrizes mais recentes disponibilizaram procedimentos claros para a sua atualização.

**Tabela 3:** Avaliação crítica das diretrizes clínicas incluídas sobre hipertensão

Autor ano	1^a^	2^b^	3^c^	4^d^	5^e^	6^f^	7^g^	8^h^	Confiança da diretriz
Barroso 2021(13)	S^i^	P^j^	P	S	S	S	S	S	Alta
Royo-Bordonada 2013 (14)	S	P	S	P	S	S	S	P	Alta
Royo-Bordonada 2016 (15)	P	N^l^	N	N	S	S	N	N	Baixa
Shah 2020 (16)	P	P	P	S	S	S	N	N	Moderada
Umemura 2019 (17)	N	P	P	P	S	S	N	N	Baixa
Unger 2020 (18)	S	S	P	S	S	S	S	S	Alta
Wang 2022 (19)	S	S	P	S	S	S	S	S	Alta
Whelton 2017 (20)	S	S	S	S	S	S	S	S	Alta

^a^ 1. Foram utilizados métodos sistemáticos para a busca de evidências?; ^b^ 2. Os critérios para a seleção de evidências estão claramente descritos?; ^c^ 3. Os pontos fortes e limitações do corpo de evidências estão claramente descritos?; ^d^ 4. Os métodos para a formulação das recomendações estão claramente descritos?; ^e^ 5. Os benefícios, efeitos colaterais e riscos à saúde foram considerados na formulação das recomendações?; ^f^ 6. Existe uma relação explícita entre as recomendações e as evidências que lhes dão suporte?; ^g^ 7. A diretriz foi revisada externamente por *experts* antes da sua publicação?; ^h^ 8. Um procedimento para atualização da diretriz está disponível?; ^i^ Sim; ^j^ Parcialmente; ^l^ Não.

As recomendações de PICS nas diretrizes clínicas para o manejo da hipertensão estão resumidas na Tabela 4. Respiração lenta guiada, ioga, mindfulness, tai chi e meditação são estratégias recomendadas para o controle da pressão arterial em indivíduos hipertensos (qualidade de evidência: moderada; força de recomendação: forte). Técnicas de relaxamento, como meditação, ioga e exercícios respiratórios melhoram o controle do estresse e da ansiedade (qualidade da evidência: moderada; força da recomendação: forte). Abordagens que promovem bem-estar e autonomia, como ioga, meditação, espiritualidade e religiosidade, destacaram-se pelo seu efeito na qualidade de vida (qualidade da evidência: moderada; força da recomendação: forte). Musicoterapia, biofeedback e grupos de autoajuda apresentam resultados promissores (qualidade da evidência: baixa; força da recomendação: fraca). 

**Tabela 4:** Síntese das recomendações de uso de PICS em diretrizes clínicas sobre hipertensão

Desfechos	Recomendações	Qualidade da evidência	Força da recomendação	Confiança
Controle da pressão arterial	Respiração lenta guiada, ioga e mindfulness têm sido recomendadas para o controle da pressão arterial em indivíduos hipertensos (13,16,18). Tai chi, ioga e meditação, bem como exercícios de relaxamento e respiração, também são indicadas como estratégias eficazes (19).	Moderada	Forte	Alta
	Meditação transcendental está associada ao melhor controle da pressão sistólica e diastólica (13). Musicoterapia também tem resultados promissores (13).	Moderada	Fraca	Alta
	Evidências insuficientes para confirmar impactos de longo prazo de ioga, meditação e biofeedback na pressão arterial (20).	Baixa	Fraca	Alta
	Medicinas complementares ou tradicionais de origem africana e chinesa não são recomendadas sem ensaios clínicos robustos (18).	Muito Baixa	Fraca	Alta
Controle do estresse e ansiedade	Meditação, exercícios respiratórios, ioga e relaxamento muscular são eficazes no controle do estresse e da ansiedade, especialmente em mulheres (14,16)	Moderada	Forte	Alta
	Ioga, meditação e biofeedback foram sugeridas para controle do estresse, embora respiração guiada e relaxamento não sejam recomendados como intervenções primárias (17,20).	Baixa	Fraca	Baixa
Qualidade de vida	Grupos de autoajuda promovem autonomia e qualidade de vida dos pacientes (15).	Baixa	Fraca	Baixa
	Espiritualidade e religiosidade foram associadas à melhora no controle da pressão arterial e aumento da qualidade de vida (13). Ioga e meditação foram recomendadas para bem-estar cardiovascular e rotina de bem-estar (19).	Moderada	Forte	Alta

## Discussão

Os achados desta revisão rápida indicam que as diretrizes clínicas sobre o manejo da hipertensão sugerem que meditação, ioga, tai chi e técnicas de respiração estão associadas à melhora do controle da pressão arterial e ao bem-estar geral dos pacientes. A análise crítica das diretrizes revelou variações quanto à clareza na formulação das recomendações e à descrição dos pontos fortes e limitações das evidências. Apesar dessas diferenças, a maioria das diretrizes mostrou-se alinhada na consideração dos benefícios e riscos à saúde, reforçando o potencial das PICS como uma abordagem complementar no manejo da hipertensão.

Entre as principais limitações desta síntese, destaca-se a restrição às diretrizes clínicas que adotaram o sistema GRADE, o que pode ter levado à exclusão de documentos relevantes que utilizam outros sistemas de avaliação. A variabilidade na qualidade metodológica das diretrizes incluídas, refletida pela inconsistência na descrição dos critérios de seleção de evidências e na formulação das recomendações, pode introduzir vieses na interpretação dos achados (21,22). A possível exclusão de diretrizes não indexadas nas bases de dados utilizadas também representa uma limitação, reduzindo o escopo da análise. O viés de publicação é uma preocupação crítica nas PICS, uma vez que estudos com resultados positivos tendem a ser publicados com maior frequência do que aqueles com achados negativos, o que pode distorcer a avaliação da real eficácia dessas intervenções (23).

Os resultados desta síntese apontam para desempenho favorável da meditação, ioga, tai chi, técnicas de respiração e mindfulness no manejo da hipertensão, evidenciando seu impacto positivo no controle da pressão arterial e na qualidade de vida dos pacientes hipertensos. Meditação para hipertensos com mais de 60 anos e ioga para hipertensos com menos de 60 anos diminuem significativamente a pressão arterial sistólica e diastólica (24). Meditação transcendental e redução do estresse baseada na atenção plena podem produzir reduções clinicamente significativas da pressão arterial (25). A base fisiológica para esses efeitos pode estar relacionada à redução do estresse e à regulação autonômica, promovida por essas práticas, o que favorece o relaxamento e a diminuição da atividade simpática, resultando em melhor controle cardiovascular (26,27). Ressalta-se que as PICS promovem um maior engajamento dos pacientes com sua própria saúde, favorecendo uma abordagem mais holística e menos medicalizada do cuidado, o que pode contribuir para a adesão ao tratamento em longo prazo (28). Tais achados reforçam a importância de considerar as PICS como uma opção viável e complementar às abordagens farmacológicas tradicionais no tratamento da hipertensão.

O potencial das PICS para promover o bem-estar e a autonomia dos pacientes hipertensos é outro aspecto relevante evidenciado nesta revisão. Intervenções como ioga e meditação mostram impacto positivo no controle da hipertensão e também melhoras na qualidade de vida e no bem-estar geral (29,30). Ao permitir que os pacientes participem ativamente no gerenciamento de sua saúde, essas práticas promovem um maior senso de autonomia e controle sobre sua condição, o que pode reduzir a dependência exclusiva de tratamentos farmacológicos (31,32). Ao favorecer o equilíbrio emocional e mental, essas intervenções podem contribuir para a redução de sintomas associados ao estresse e à ansiedade, fatores frequentemente correlacionados com a hipertensão (33,34). Essa abordagem mais integrada ao tratamento, ao envolver aspectos físicos, mentais e espirituais, alinha-se com um modelo de atenção centrado no paciente, promovendo não apenas o alívio dos sintomas, mas uma transformação no estilo de vida que beneficia tanto a saúde física quanto o bem-estar psicológico.

Estudos futuros devem realizar avaliações clínicas mais rigorosas e padronizadas sobre as PICS na hipertensão arterial. Uma revisão sistemática evidenciou que as diretrizes clínicas atuais mencionam pouco essas práticas, destacando uma lacuna importante para orientar profissionais e pacientes (35). Sugere-se que novas diretrizes clínicas utilizem métodos sistemáticos e transparentes, preferencialmente o sistema GRADE, para melhorar a qualidade das recomendações. No Brasil, estudos acadêmicos indicam efeitos positivos de técnicas como ioga, acupuntura e meditação na redução da pressão arterial, porém reforçam a necessidade de métodos mais robustos e amostras maiores (36). Assim, recomenda-se que pesquisas futuras adotem delineamentos rigorosos, como ensaios clínicos randomizados, com amostras representativas. Estudos também devem investigar o impacto das PICS em desfechos de longo prazo, como adesão ao tratamento, qualidade de vida e custo-efetividade, visando fornecer evidências abrangentes e sustentáveis para a prática clínica.

Em conclusão, esta revisão rápida aponta que as PICS apresentam benefícios no manejo da hipertensão, tanto no controle da pressão arterial quanto na melhora da qualidade de vida dos pacientes. Embora haja variações na qualidade metodológica das diretrizes, as evidências indicam que intervenções como meditação, ioga, tai chi e mindfulness são estratégias eficazes e seguras como complemento ao tratamento convencional. Esses achados reforçam a relevância das PICS como uma abordagem promissora no cuidado integral da hipertensão, com potencial para melhorar o bem-estar e a autonomia dos pacientes.

## Data Availability

Não houve criação de banco de dados e/ou códigos de análise nesta pesquisa, além das ilustrações já disponíveis no manuscrito.
